# Impact of the COVID-19 Outbreak on Orthopedic Surgery: A Nationwide Analysis of the First Pandemic Year

**DOI:** 10.7759/cureus.17252

**Published:** 2021-08-17

**Authors:** Maximiliano Barahona, Carlos A Infante, Miguel J Palet, Macarena A Barahona, Cristian Barrientos, Alvaro Martinez

**Affiliations:** 1 Orthopaedic Department/Knee Surgery, Clinica Bupa Santiago, La Florida, CHL; 2 Orthopaedic Department, Hospital Clinico Universidad De Chile, Santiago, CHL; 3 Orthopaedic Department, Hospital Clínico de la Universidad de Chile, Santiago, CHL

**Keywords:** orthopaedic surgery, elective surgery, covid-19, mobility, pandemic, coronavirus

## Abstract

Purpose

To analyze the impact of the coronavirus disease 2019 (COVID-19) outbreak during the first pandemic year in a single country.

Methods

A cross-sectional study was designed. The free access database of the Chilean Department of Statistics and Health Information (DEIS) was used to compare the number of orthopedic procedures between 2019 and 2020. Country mobility was exported from the Institute of Complex Engineering Systems (ISCI) free-access database; this corresponds to a direct measurement of the degree of confinement of the country. Spearman correlation (rho) was used to analyze the total monthly COVID infection trend and mobility to orthopedics procedures.

Results

The number of orthopedic surgeries fell by 22.8% during the first year of the pandemic. All surgical procedures were adversely affected, with the fracture/trauma surgeries being the least affected. The maximum adverse impact was seen in knee arthroplasty (-64%), followed by hip arthroplasty (-41%) and knee ligament reconstruction (-44%). The number of orthopedic procedures had a mild correlation to the monthly number of COVID-19 cases (rho=-0.53, p=0.08) and a strong correlation with the country's mobility (rho=0.94, p=0.0001).

Conclusions

The COVID-19 outbreak diminished the number of orthopedic procedures during 2020, and the impact was directly correlated to the country's mobility. The public health network did have a more significant adverse impact in elective surgeries due to a slower recovery than private institutions. An increase in the waiting list should be expected, which will widen the difference in access to orthopedic surgery in Chile.

## Introduction

The first case of severe acute respiratory syndrome coronavirus 2 (SARS-CoV-2) was reported in China; after which the novel coronavirus spread out to the rest of the world, creating a global health emergency that led to the World Health Organization (WHO) declaring it a pandemic on March 11, 2020 [1].

The pandemic has caused more than three million deaths and led governments to promote social distancing and dictate lockdown measures to stop the spread and reduce mortality. The coronavirus response has disrupted the health care system globally, leading to the suspension of elective or non-essential surgeries [2-3].

The first case declared in Chile was on March 3, 2020. The central government took several measures to slow down the pandemic spread, such as nationwide curfew and cancellation of events, shopping malls, schools, and universities [4]. Also, conversion of hospital beds and suspension of elective surgeries were decreed to increase the availability of intensive care units [5]. This had a direct impact on the number of orthopedic procedures performed in our country.

In Chile, two types of institutions provide inpatients services: the public health network (PHN) and private health institutions (PHI). A recent study showed that orthopedic procedures have grown in the last 16 years in both types of institutions. The same survey reported that the most frequent orthopedic procedures performed in Chile between 2004 and 2019 were: knee arthroscopy, rotator cuff repair, knee arthroplasty (KA) and hip arthroplasty (HA), knee ligament reconstruction, and hip, wrist, ankle, and open fractures. Those procedures accounted for 40% of the total orthopedic procedures [6].

This study aims to analyze the impact of the coronavirus disease 2019 (COVID-19) outbreak during the first pandemic year using a nationwide database to compare the total number of surgeries between 2019 and 2020. We hypothesize that the overall number of procedures and the most prevalent surgeries experienced a decrease in 2020 compared to 2019, with the second trimester and public institutions being the most affected, as it corresponds to the period of greatest confinement in Chile. Also, our theory is that the number of orthopedic procedures will strongly correlate with the monthly number of COVID-19 cases and the country's mobility.

## Materials and methods

A cross-sectional study was designed. Our institution's ethics committee board declared that the present study does not require approval since the data analyzed correspond to free open-access data.

The free access database of the Chilean Department of Statistics and Health Information (DEIS) depends directly on the Ministry of Health and stores all hospital discharges records of the country. The databases from 2019 and 2020 were downloaded from the DEIS homepage (https://deis.minsal.cl/#datosabiertos). The software program Microsoft Access (Microsoft Corporation, Redmond, WA) was used to manage the data.

All patients that underwent orthopedic surgery between January 1, 2019, and December 31, 2020, were included in this analysis. The incidence rate of surgeries was calculated per 100,000 inhabitants (IR) using the population reported by the National Statistics Institute of Chile (INE). Country mobility was exported from the Institute of Complex Engineering Systems (ISCI) free access database, which analyzed country mobility using mobile phone global positioning system (GPS) data. Country mobility measures the movement within and between cities carried out by the population, meaning that it is a direct indicator of the degree of confinement in the country. The reference used was the first two weeks of March 2020; therefore, mobility is expressed in the percentage of change of those two weeks, for example, a mobility of -0.15 means that during that month, mobility was 15% less than the first two weeks of March 2020. The database is available at https://covidanalytics.isci.cl/movilidad.

First, an exploratory analysis was conducted. Then, Spearman's correlation (rho) was used to analyze the total monthly COVID infection trend and mobility to orthopedics procedures. A 5% significance was used. Data were exported to STATA v.17 (StataCorp LP, College Station, Texas, USA) for statistical analysis.

## Results

A total of 1,330,477 hospital discharges were identified in 2020, of which 99,333 were orthopedic surgical procedures (7.47%). Compared to 2019, the overall reduction in the total hospital discharges was 19% (n=1646680), and the orthopedic surgical work reduced by 22.8% (n=128735). The incidence rate (IR) in 2019 was 687.4 procedures per 100,000 inhabitants and fell to 510.5 in 2020 (Table 1).

**Table 1:** Summarise the number of total hospital discharges (TD), orthopedic discharges (OD), and the incidence rate (IR) of orthopedic surgeries per 100,000 inhabitants in Chile since 2015 Abbreviations: TD=total discharges; OD=orthopedic discharges; IR=incidence rate per 100000 inhabitants

Year	TD	OD	OD/TD	IR
2020	1330477	99333	7.47%	510.5
2019	1646680	128735	7.82%	687.4
2018	1669602	127321	7.63%	686.3
2017	1637150	118766	7.25%	646.4
2016	1637265	113662	6.94%	624.8
2015	1671054	109778	6.57%	609.7

Age and gender remained similar between 2019 and 2020. The mean age was 48.0 (standard deviation: ±21.3) in 2020 and 47.6 (standard deviation: ±21.3) in 2019. Men were more prone to orthopedic surgical procedures. During 2020, 58.15% were performed in men and 56.61% in 2019.

All surgical procedures were adversely affected in 2020. Knee replacement (-64%), hip replacement (-41%), knee ligament reconstruction (-44%), knee arthroscopy (-36%), and cuff rotator repair (-30%) were the surgical procedures that were above the overall reduction in orthopedic procedures. All fracture procedures were least affected, reaching -14% for all surgical fractures, -11% for hip fracture, -4% for wrist fracture, and -10% for complete treatment of an open fracture (Table 2).

**Table 2:** The incidence rate (IR) of orthopedic procedures per 100,000 inhabitants performed in 2019 and 2020 in Chile *Above the decrease of total procedures compared to 2019

IR	2020	2019	Dif
Knee arthroscopy	51.52	82.62	-36%*
Cuff repair	24.94	35.39	-30%*
Hip arthroplasty	29.62	50.20	-41%*
Knee arthroplasty	10.13	28.23	-64%*
Knee ligament	11.49	20.50	-44%*
Fractures	212.67	248.11	-14%
Hip fracture	32.89	36.77	-11%
Ankle fracture	34.42	39.83	-14%
Wrist fracture	21.89	22.74	-4%
Open fracture	18.29	20.39	-10%
Other surgery	308.20	384.27	-19%

**Figure 1:**
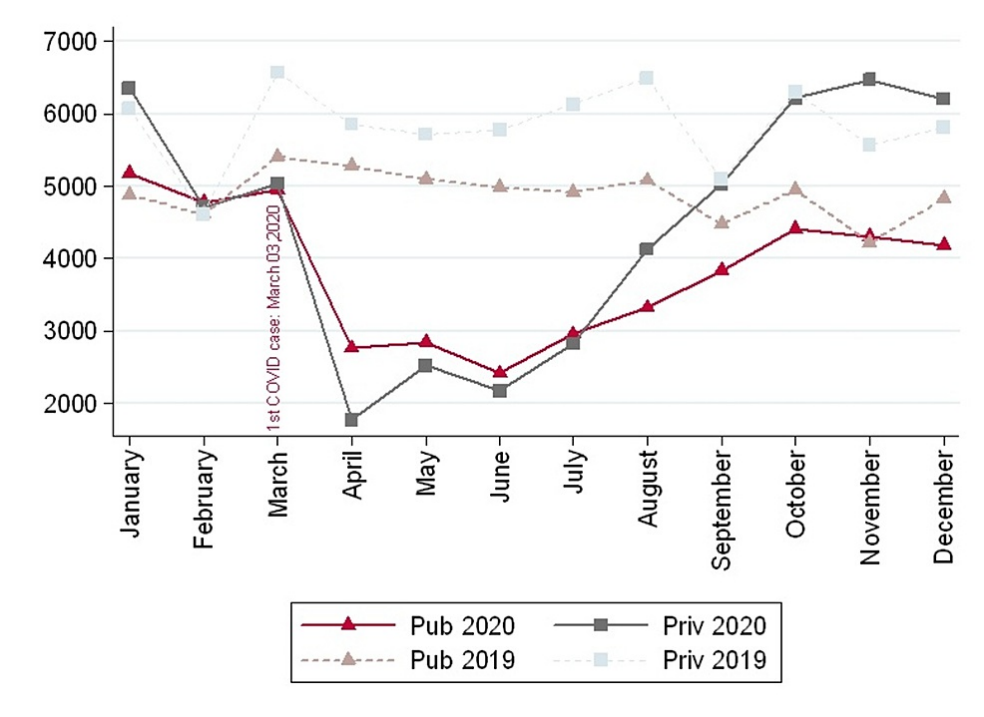
Comparison of total procedures between 2019 and 2020 by types of institutions in Chile

Private institutions performed 53,418 surgeries and public institutions 45,915, which accounted for a 24% and 22% reduction, respectively, as compared to 2019 (Table 3). The trend during 2019 and 2020 in both institutions is shown in Figure 1. Hip and knee arthroplasty went down in both institutions, but the impact was more significant in the public health network, achieving a decrease of 28% in HA and 11% in KA more than in private institutions. The same trend was observed in knee arthroscopy, rotator cuff repair, and knee ligament reconstruction, reaching a 37%, 45%, and 9% decrease in public institutions. On the other hand, the total number of orthopedic and fracture procedures were the least affected in the public network, reaching 2% and 11% less than in private institutions.

**Table 3:** The number of orthopedic procedures performed by types of institutions in 2019 and 2020 *Above the decrease of total procedures compared to 2019 Abbreviations: Public=public health institutions; Private=private health institutions

	2020 Public	2019 Public	Difference	2020 Private	2019 Private	Difference
Knee arthroscopy	660 (6.58%)	1934 (12.81%)	-66%*	9365	13163	-29%*
Cuff repair	460 (9.48%)	1253 (18.90%)	-63%*	4392	5375	-18%
Hip arthroplasty	2914 (50.56%)	5786 (50.56%)	-50%*	2849	3615	-21%
Knee arthroplasty	961 (48.73%)	2963 (56.04%)	-68%*	1011	2324	-56%*
Knee ligament	153 (6.84%)	305 (7.94%)	-50%*	2083	3534	-41%*
Fractures	25262 (55.02%)	27091 (46.13%)	-6%	16120	19371	-17%
Hip fracture	4973 (10.83%)	5390 (9.18%)	-8%	1427	1495	-5%
Ankle fracture	3960 (59.13%)	4320 (57.92%)	-8%	2737	3138	-13%
Wrist fracture	2029 (47.64%)	2521 (48.05%)	-20%	2230	2726	-18%
Open fracture	1714 (48.16%)	1834 (48.04%)	-7%	1845	1984	-7%
Other surgery	33064 (55.13%)	37814 (52.55%)	-13%	26906	34146	-21%
Total	45915 (46.22%)	58730 (45.62%)	-22%	53418	70005	-24%

The months of the lower number of surgeries correspond to April, May, June, and July, and private institutions managed to make a faster recovery, reaching 2019 levels in September 2020. In contrast, it took two more months for public health centers.

**Figure 2:**
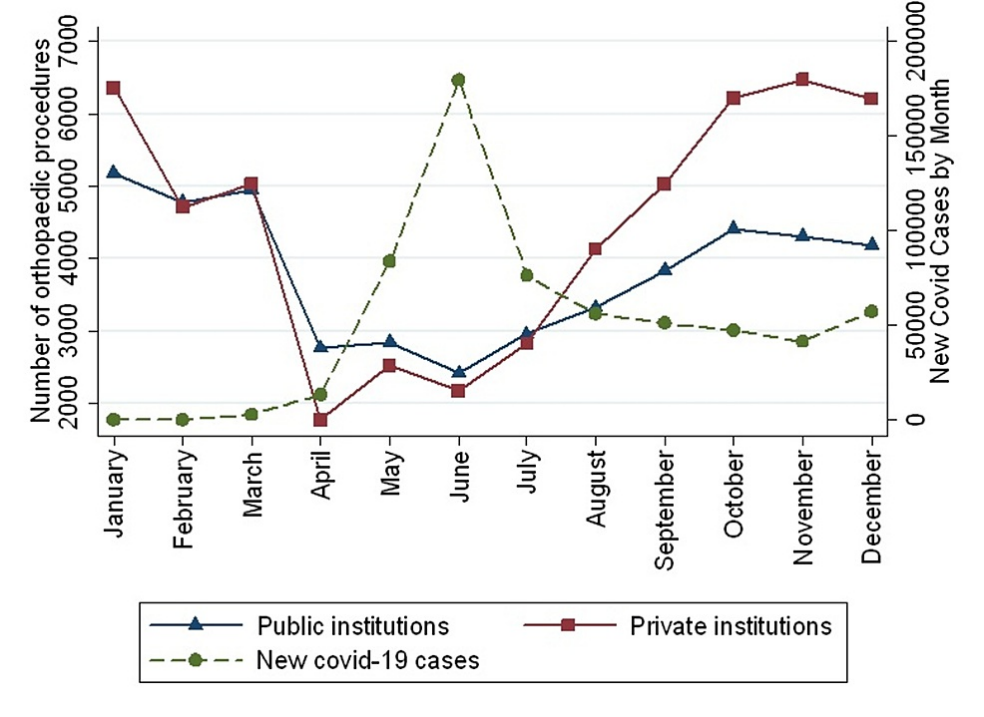
The number of orthopedic procedures by institutions and the number of new COVID-19 reported monthly by the Chilean Minister of Health

There is a mild correlation between the total cases of COVID-19 reported monthly to the number of orthopedic procedures (rho=-0.53, p=0.08). Nevertheless, the number of orthopedic surgeries in public institutions showed a strong correlation with the number of COVID-19 patients (rho=-0.76, p=0.004) (Figure 2).

**Figure 3:**
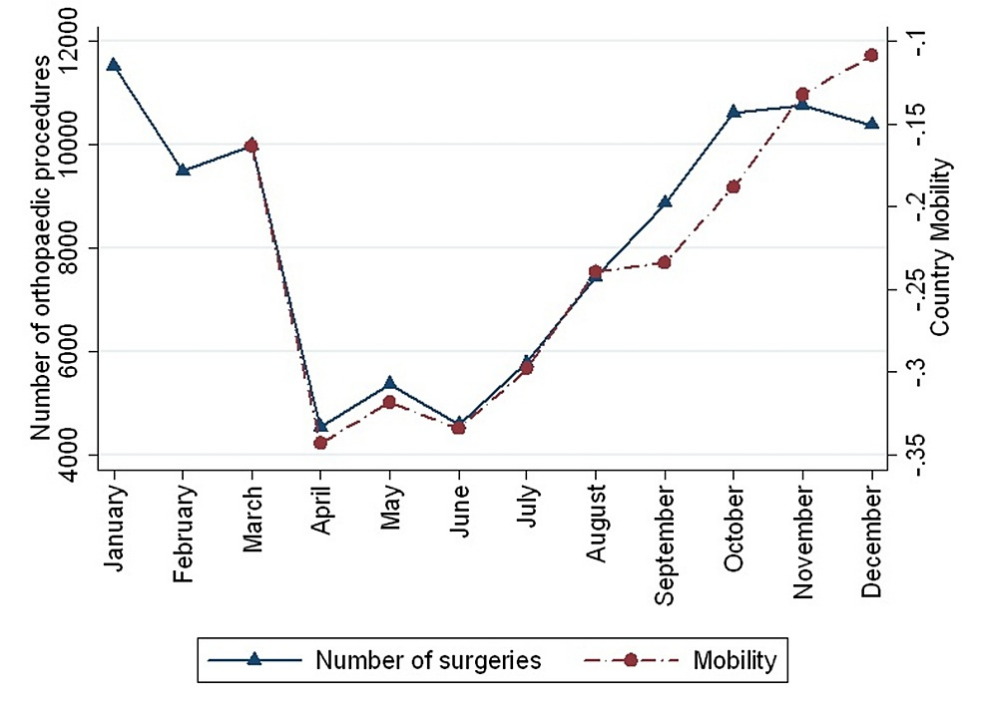
Relation between the total number of procedures and country mobility during 2020

**Figure 4:**
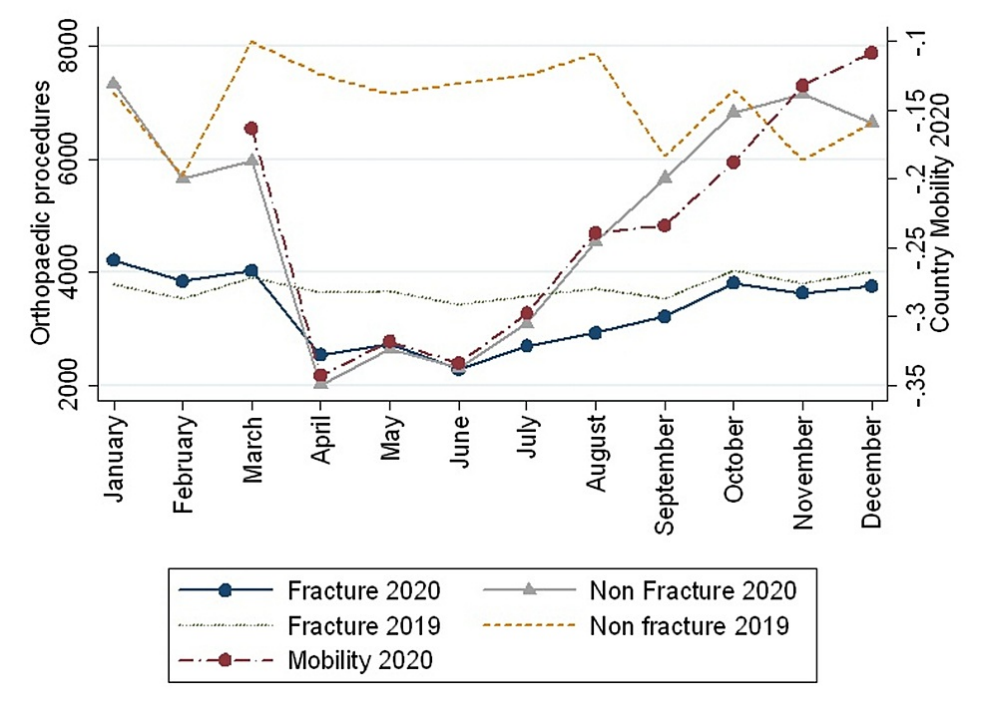
The number of fracture and non-fracture procedures between 2019 and 2020 and its relations with the 2020 country mobility is shown

Country mobility showed a strong correlation with the number of orthopedic surgeries (rho=0.94, p=0.0001) (Figure 3). The number of surgeries carried out in public and private institutions was also strongly correlated with city mobility, reaching a rho of 0.88 (p=0.0008) and 0.94 (p=0.0001), respectively. Also, mobility had a strong correlation with fractures surgeries (rho=0.88, p<0.000) and non-fractures procedures (rho=0.94, p=0.0001). Despite the increasing mobility, fracture procedures did not increase after September 2020, whereas non-fracture procedures keep growing according to the country's mobility (Figure 4).

## Discussion

The main finding in this study was that the COVID-19 outbreak reduced the number of surgeries in Chile as compared to 2019 by 22.3%. The most affected months were May, June, and July, which according to the country mobility was the period of greatest confinement. A study from India performed in a single center reported a reduction of 43.92% in orthopedic surgeries during a whole year [7]. To our knowledge, this is the first nationwide research showing the impact of COVID-19 on orthopedic surgery during an entire year.

Country mobility had a strong correlation with the number of procedures; also, the number of COVID-19 cases had a more significant impact on the number of procedures performed in the public health network. Mobility restrictions dictated by the Chilean government were determined by factors like accumulated COVID-19 cases, hospital capacity, and socioeconomics [8]. The fear of contracting the infection also made the population decide to reduce the number of trips, especially in the first months after the first case of COVID-19 was reported [8]. Mobility measured by GPS phones has been linked to the growth in COVID-19 cases and predicts the pandemic trend [9-10]. The strong correlation between mobility and orthopedic procedures found in this study was probably linked to the hospital availability of operating theaters and the likelihood of a traumatic injury. However, as the fractures procedures resume the 2019 trend early, it seems more likely that the hospital capacity is more crucial. Also, many orthopedic surgeons have been redeployed to assist in wards, managing COVID-19 patients and performing administrative tasks to support their health institutions during the peak months, with the consequent reduction of orthopedic procedures [11-14].

During the second wave in Japan, Nakai et al. [15] showed that orthopedic procedures performed in patients who tested negative before surgery did not raise the rate of complications, even in those who were immunoglobulin G (IgG) positive. These findings are supported by an observational study in the United Kingdom (UK), in which no significant complications were found in lower limb trauma surgery during the peak of COVID-19 [16]. Testing for COVID-19 before surgery is essential to reduce risk, even in asymptomatic patients [17]. Moreover, Kader et al. estimated a low mortality risk when performing elective surgeries in asymptomatic patients when all of them are tested for COVID-19, even with the risk of having a false negative test [18]. Hence, every hospital institution should weigh the benefits of performing elective surgery based on personnel, hospital availability, PPE supply, and state policy [19-20]. 

A critical pathology in orthopedics is hip fracture, which needs interdisciplinary care to improve outcomes and survival [21]. Reports from the UK show an increased 30-days mortality after hip fracture during the first wave of the outbreak [3,22], with the risk of mortality being higher in COVID-19-positive patients [23]. This research shows a decrease in hip fracture surgeries. A lower incidence rate may play a role, however, it is unlikely, as other countries report a reduction in quality care in this pathology [24], and no incidence rate reduction was noted during the first pandemic wave [25]. Hip fracture is related to fragility, so inactivity in older people should increase sarcopenia and osteoporosis; the latter should also be affected by confinement, as exposure to the sun is related to vitamin D metabolism [26-28]. So, presumably, the incidence of hip fracture remained still, and Chile had shown recently to be deficient in hip fracture treatment [29]. Efforts to keep hip fractures units functioning should be made to not increase this vulnerable group of patients' morbidity, mortality, and long-term healthcare costs [24].

The demands for elective surgery will increase between pandemic waves; therefore, health systems should be prepared to prioritize patients [30]. Before the outbreak, the incidence of knee and hip replacement in Chile was the second last between OCDE countries [31], so the decrease of 41% and 61% during 2020 means that the gap has increased as other countries reported only a 16% decrease [32]. The waiting list for these procedures will put pressure in the future years on the health system, as patients will still need their surgery to improve their quality of life [33]. At the moment, prioritization to elective surgery in Chile has been made using as criteria the diagnosis of cancer and that the pathology belongs to the Explicit Health Guarantees (GES) - a set of benefits guaranteed by Chilean law allowing access, opportunity, financial protection, and quality of care in a designated list of diseases). The only orthopedic surgery in Chile that belongs to GES is hip replacement in patients above 65 years, so all other procedures are at risk of not being prioritized.

The less marked recovery after the first wave of COVID-19 in public institutions should raise more concerns. Only non-fracture procedures had a lower decrease in public than private institutions; moreover, the public health network took two months more than private institutions to recover the trend of 2019. This means that many surgeries have been postponed and will lengthen the waiting list, increasing the pressure on the public health system. Moreover, during 2021, Chile has presented two new waves (March and June). The impact of them remains to be seen; however, according to the results of this research, a more significant number of postponed orthopedic procedures is expected. This only will broaden the gap of access to orthopedic surgery that already exists [6]. A prediction made in the UK in November 2020 suggests that if the level of productivity increased to 30%, it would take 20 to 46 months to return to the pre-COVID-19 waiting list number [34]. This implies that patients who require surgery to improve their quality of life should be supported in obtaining access to it following rigorous protocols to ensure a free COVID-19 environment [35].

COVID-19 has exhibited and increased social inequalities worldwide and in particular in Latin America [36]. The impact of orthopedic surgeries is another aspect in which this becomes evident. This is even more worrying for Chile because the socioeconomic differences, including health care inequities, have already caused social protest in the country in 2019 [37].

The COVID-19 outbreak has stressed all systems worldwide, and restoring orthopedic surgery rates to that pre-COVID-19 era will be challenging [38]. The results reported in this study are essential to understand the magnitude of the pandemic, especially in the public network, and to help policymakers and health care providers plan health policies related to orthopedic procedures. 

The limitations of this study are related to the lack of national registries of any orthopedic procedures in our country. This national open-access database registers all the procedures performed in the country regarding the type of institution or insurance, race, or socioeconomic or geographic factors but does not store any data about complications or follow-up, so it was not possible to weigh the impact of the coronavirus outbreak in the mid or long term outcomes after the procedures. Another limitation is that some procedures performed by orthopedic surgeons could have been registered under a neurosurgery-related code, as lumbar spine surgery or median nerve neurolysis. The former is a significant reason why Chile urgently requires modernization of the surgical coding system and national registries to allow follow-up after a surgical procedure. Nevertheless, this bias should affect both years equally.

Additionally, concerns about extrapolating these results to other countries may not be applicable, as each country took different measures of confinement and had a heterogeneous incidence of covid cases; however, the strong correlation found between the country's mobility and the number of surgeries may be the way to predict the number of orthopedic surgeries performed in other countries.

## Conclusions

The COVID-19 outbreak diminished the number of orthopedic procedures during 2020 as compared to 2019. The maximum adverse effect was during the second trimester, which according to country mobility, was the period of greatest confinement. Nevertheless, the last trimester showed a recovery, reaching similar numbers to 2019. The public health network did have a more significant adverse impact on elective surgeries due to slower recovery than private institutions. An increase in the waiting list should be expected, which will widen the difference in access to orthopedic surgery in Chile.
